# The relationship of 1,25-dihydroxyvitamin D and Vitamin D binding protein in periodontitis

**DOI:** 10.12669/pjms.35.3.482

**Published:** 2019

**Authors:** Sara Rafique, Mozaffer Rahim Hingorjo, Mahparah Mumtaz, Masood Anwar Qureshi

**Affiliations:** 1 *Dr. Sara Rafique, MPhil, Department of Physiology, Jinnah Medical & Dental College, Karachi, Pakistan*; 2 *Prof. Mozaffer Rahim Hingorjo, PhD, Department of Physiology, Jinnah Medical & Dental College, Karachi, Pakistan*; 3 *Dr. Mahparah Mumtaz, BDS, Department of Physiology, Jinnah Medical & Dental College, Karachi, Pakistan*; 4 *Prof. Masood Anwar Qureshi, PhD, Department of Physiology, Dow University of Health Sciences, Karachi, Pakistan*

**Keywords:** Periodontitis, Vitamin D, Vitamin D binding protein

## Abstract

**Objective:**

This study was conducted to explore the relationship between 1,25-dihydroxy vitamin D (1,25(OH)_2_D) and Vitamin-D binding protein (DBP) in patients with periodontitis and healthy controls.

**Methods:**

Seventy-five periodontitis cases were recruited from the dental OPD of Dow University of Health Sciences, Karachi. Diagnostic criteria of periodontitis were followed according to the probe pocket depth and clinical attachment loss. Seventy-five periodontal healthy controls were selected from the faculty and students of same university. Serum levels of 1,25(OH)_2_D and DBP were determined by ELISA.

**Results:**

Significantly low levels of 1,25(OH)_2_D and high levels of serum DBP were observed in periodontitis patients compared to healthy controls (*p*<0.05), with levels of DBP increasing significantly with the severity of periodontitis (*p*=0.005). Concentrations of DBP correlated positively with 1,25(OH)_2_D, especially in cases with periodontitis (r =0.780; p<0.001).

**Conclusion:**

Within the limits of the study, we conclude that low 1,25(OH)_2_D levels and high DBP levels are associated with periodontitis.

## INTRODUCTION

Periodontitis is inflammation of the supporting tissues of teeth resulting in gingival detachment and disintegration of alveolar bone.^1^ This is a painless disease presenting with features of tooth mobility and bleeding from gingiva. Prevalence of periodontitis in adults globally is 5-20% with higher prevalence seen in Pakistan.^2^,^3^ Recent studies have shown strong association of periodontitis with systemic diseases such as cardiovascular disease, diabetes mellitus Type-2 and osteoporosis.^4^

1,25 dihydroxy vitamin D (1,25(OH)_2_D), apart from regulating calcium homeostasis, plays an important role in immune modulation, boosting the immune system by upregulating the production of antimicrobial peptides. Of these, the cathelicidins have an increasingly broad spectrum of action against both bacteria and viruses. Vitamin D also dampens the arm of the immune system that is invested in order to cause inflammation.^5^

Vitamin D binding protein (DBP) is the major transporter of the two forms of vitamin D, 25(OH)D and 1,25(OH)_2_D. By binding the hormone, it plays an essential role in regulating the free hormone level. Of the two forms of vitamin D, 25(OH)D or calcidiol binds to a larger extent with DBP. Vitamin D binding protein prevents excretion of 25(OH)D in the urine by facilitating receptor mediated endocytosis from the proximal tubule via megalin receptor. This receptor is present on the luminal membrane of the tubular epithelium and is responsible for internalization of DBP-25(OH)D complex inside the cell. The complex is intracellularly cleaved by lysosomes and 25(OH)D released. It is converted to 1,25(OH)_2_D by renal 1-α hydroxylase.^6^

Vitamin D binding protein also regulates bone homeostasis by its effect on osteoclasts. Like 1,25(OH)_2_D, DBP also modulates immune function playing important roles in both acute and chronic inflammation. It is an acute phase reactant protein and its levels are elevated up to 20% in acute inflammation.^7^ Recent studies have found DBP to be widely distributed in periodontal tissues where it is highly expressed, thereby speculating its important role in local immune defense.^8^

There are few studies reporting the role of 1,25(OH)_2_D and DBP in periodontitis. In this study, we explored the relationship between 1,25(OH)_2_D and DBP in patients with periodontitis and healthy controls.

## METHODS

This was a case-control study conducted at Dow University of Health Sciences (DUHS), Karachi, Pakistan. Seventy-five subjects aged 18-40 years, having periodontitis were recruited from dental OPD. Periodontitis was diagnosed by identifying Clinical Attachment Loss (CAL) ≥1mm at two or more nonadjacent sites. Further grouping into mild, moderate, and severe was done based on CAL 1-2mm, 3-4mm, and ≥5mm, respectively. Healthy controls (n=75) were selected from students and faculty of DUHS, identified as those with PPD ≤ 3 mm, no bleeding on probing, no clinical attachment loss and no radiographic evidence of bone loss. Excluded were those with systemic disease, periodontitis involving wisdom teeth, periodontal therapy in the last year, and those receiving vitamin D and calcium supplementation. Written informed consent was taken from subjects and the study was approved by the institutional review board of DUHS.

Besides PPD and CAL, oral examination included assessment of gingival, plaque, calculus and mobility indices. In addition to a dental exam, bitewing radiographs of the subjects were also taken. Biochemical analysis was performed for serum 1,25(OH)_2_D and DBP using ELISA, having sensitivity to detect lowest level of 1,25(OH)_2_D = 2 pg/mL and DBP = 8 ug/mL.

Descriptive statistics were used to evaluate the characteristics of each participant. Independent sample t-test was performed to test differences between 1,25(OH)_2_ D and DBP among healthy controls and periodontitis. Pearson’s correlation coefficient was used to determine the association of serum 1,25(OH)_2_ D with DBP among the study participants, cases and controls. The significance level was taken at p<0.05. Statistical analysis was done using SPSS statistics (version 23.0).

## RESULTS

A total of 150 subjects (44% males, 56% females) including 75 patients with periodontitis and 75 healthy controls were enrolled in this study. The mean age of participants was 31.23±2.70 years and was not significantly different between the two groups. Of the total number of cases, mild, moderate and severe periodontitis was seen in 12%, 13.3% and 74.6%, respectively. The baseline characteristics of the study population are shown in Table-I. The oral health indices including gingival index, plaque index, calculus index, mobility index, CAL and PPD, were significantly higher in cases (all P_trend_ <0.001). Compared to controls, cases with periodontitis had significantly low levels of 1,25(OH)_2_D and high levels of DBP (*p*-value 0.023 and < 0.001, respectively).

**Table-I:** Descriptive Characteristics of Study Population (n=150).

Parameters	All (n = 150)	Cases (n = 75)	Controls (n = 75)	p-value
***General Characteristics***				
Age, years	31.23±3.70	31.60±4.38	30.85±2.86	0.21
Gender, n (male/female)	150 (66/84)	75 (37/38)	75 (29/46)	0.18
***Oral Health Indices***				
Gingival Index	0.854±0.749	1.45±0.566	0.250±0.265	<0.001
Plaque Index	0.654±0.646	1.04±0.697	0.264±0.219	<0.001
Calculus Index	0.684±0.574	1.09±0.540	0.277±0.192	<0.001
Mobility Index, mm	0.940±1.01	1.88±0.518	0	<0.001
CAL, mm	2.21±2.34	4.41±1.07	0	<0.001
PPD, mm	2.30±1.61	3.50±1.00	1.10±1.15	<0.001
***Biochemical Parameters***				
1,25(OH)_2_D, pg/ml	40.90±48.42	31.94±32.75	49.86±59.04	0.023
DBP, ug/ml	73.93±92.74	99±111.53	48.86±60.05	<0.001

***Abbreviations:*** **MI:** Mobility Index, **CAL:** Clinical attachment loss, **PPD:** Periodontal probing depth, 1,25(OH)2D, 1,25-dihydroxyvitamin D, **DBP:** Vitamin D binding protein.

The results of one-way ANOVA to see the effect of serum 1,25(OH)_2_D and DBP upon the severity of periodontitis are shown in Table-II. The mean DBP levels in mild periodontitis were 65.33±91.90 ug/mL and significantly increased along with the severity of periodontitis (*p*=0.005). Serum 1,25(OH)_2_D, however, did not change significantly with the severity of periodontitis.

**Table-II:** Comparison of 1,25(OH)_2_D & Vitamin D Binding Protein with Severity of Periodontitis

Parameters	Mild (n = 9)	Moderate (n = 10)	Severe (n = 56)	p value
1,25(OH)_2_D, pg/mL	23.16±28.79	26.76±17.62	34.27±35.34	0.128
DBP, ug/mL	65.33±91.90	97.73±98.97	104.63±117	0.005

***Abbreviations:*** 1,25(OH)2D, 1,25-dihydroxyvitamin D, **DBP:** Vitamin D binding protein.

***Note:*** One-way ANOVA was used to compare means between cases. **p<0.01= very significant Periodontitis was classified on the basis of Clinical Attachment Loss.

**Fig.1:**
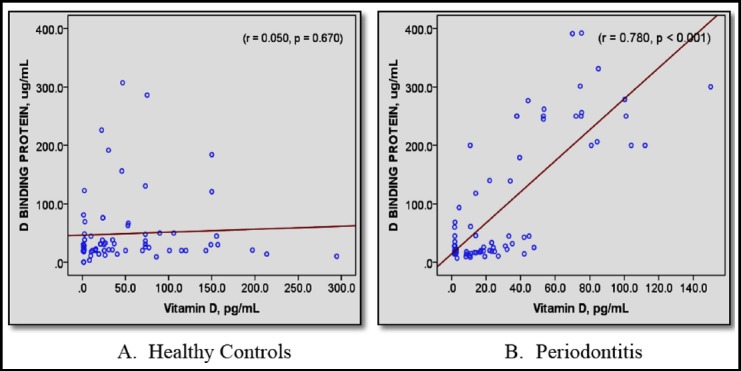
Scatter plot showing Pearson’s correlation (*r*) between serum DBP (ug/mL) and 1,25(OH)_2_D (pg/mL). *p* < 0.05 termed significant.

A significant positive correlation was observed between DBP and 1,25(OH)_2_D in subjects with periodontitis (*r*=0.78; *p*<0.001) but no such correlation was observed in healthy controls (Fig.1).

## DISCUSSION

In the present study, the association of periodontitis was explored with serum levels of 1,25(OH)_2_D and DBP. Vitamin D plays an important role in maintaining a healthy periodontium by preventing bone loss and alleviating inflammation.^9^ We observed low levels of 1,25(OH)_2_D in patients with periodontitis, which is in agreement with other studies.^10^,^11^ This supports the hypothesis provided for the inverse association of vitamin D with periodontitis. This states that high levels of vitamin D, due to their immunomodulatory properties, prevent inflammations such as periodontitis. Formation of 1,25(OH)_2_D within macrophages promotes the translation of a bactericidal agent, cathelicidin, the only known human protein having bactericidal properties.^6^ Low serum levels of 1,25(OH)_2_D observed in the present study maybe due to decreased conversion from its less active form or increased degradation. It could also be due to less accessibility of free 1,25(OH)_2_D, being bound to the increased number of immune cells found in periodontitis, with increased consumption of the hormone by immune cells to combat against periodontal pathogens.^12^ However, vitamin D deficiency should be considered a relative or functional deficiency and not just some laboratory value as the action of vitamin D is dependent on its receptor, which is genetically regulated, resulting in variations in effective dose and cutoff levels of vitamin D in different populations.

We also observed elevated DBP levels in cases as compared to controls, highlighting their role in inflammation. Moreover, there was a gradual rise in DBP levels from healthy state to periodontitis which is in agreement with other studies.^13^ Being an acute phase reactant protein, DBP is elevated systemically in plasma during the inflammatory process. Furthermore, being a major transporter of vitamin D, it has direct as well as indirect immunomodulatory functions. During inflammation there is increased affinity for the neutrophils to DBP, augmenting the action of C5a.^14^ It also promotes the formation of DBP-macrophage activating factor that enhances phagocytic properties of macrophages by generation of superoxide. Vitamin D binding protein is also seen to enhance activation of osteoclasts promoting the bone resorption seen in periodontitis.^15^,^16^

Similar to our study, the association of DBP with periodontal health and disease has been observed by Krayer JW et al. showing DBP levels to be significantly raised in the parotid saliva of periodontitis subjects.^17^ Zhang et al in 2014 published a case control study comparing DBP levels in plasma with gingival crevicular fluid (GCF). The results showed decreased DBP levels in GCF compared to plasma which may have been due to increased consumption of DBP in the inflammatory state. In addition to GCF, DBP has also been found in the periodontium apparatus, which is another medium that participates in the modulation of periodontitis.^18^

The current study also showed a positive association of DBP with 1,25(OH)_2_D. However, this was true for subjects with periodontitis only, having active inflammation. This may be due to the fact that DBP provides increased amount of 25(OH)D to the kidney to be converted into 1,25(OH)_2_D. The activated vitamin D is then consumed by the immune cells while combating periodontal pathogens.^10^

## CONCLUSION

Periodontitis subjects had elevated levels of DBP while having low serum 1,25(OH)_2_D. The levels of DBP increased significantly along with the severity of periodontal destruction and may be used as a biomarker to complement the diagnosis of periodontitis in early as well as late stages. Addition of 1,25(OH)_2_D to the treatment of periodontitis may be reflected in DBP levels and needs to be explored further in order to prescribe vitamin D for treatment and prevention of periodontitis.

### Authors’ Contribution

**SR, MRH &MM:** Conceived, designed and did statistical analysis & editing of manuscript.

**SR, MRH, MM & MAQ:** Did data collection and manuscript writing, did review and final approval of manuscript.
